# Deciphering the Role of Humoral and Cellular Immune Responses in Different COVID-19 Vaccines—A Comparison of Vaccine Candidate Genes in Roborovski Dwarf Hamsters

**DOI:** 10.3390/v13112290

**Published:** 2021-11-16

**Authors:** Jakob Trimpert, Susanne Herwig, Julia Stein, Daria Vladimirova, Julia M. Adler, Azza Abdelgawad, Theresa C. Firsching, Tizia Thoma, Jalid Sehouli, Klaus Osterrieder, Achim D. Gruber, Birgit Sawitzki, Leif Erik Sander, Günter Cichon

**Affiliations:** 1 Institute of Virology, Freie Universität Berlin, 14163 Berlin, Germany; jakob.trimpert@fu-berlin.de (J.T.); daria.vladimirova@fu-berlin.de (D.V.); Julia.adler@fu-berlin.de (J.M.A.); azza.abdelgawad@fu-berlin.de (A.A.); 2 Department of Gynecology, Charité Universitätsmedizin Berlin, Campus Virchow Klinikum, 13353 Berlin, Germany; Susanne.herwig@charite.de (S.H.); Jalid.sehouli@charite.de (J.S.); 3 Institute of Medical Immunology, Charité Universitätsmedizin Berlin, 13353 Berlin, Germany; Julia.stein@charite.de (J.S.); Tizia.thoma@charite.de (T.T.); Birgit.sawitzki@charite.de (B.S.); 4 Institute of Veterinary Pathology, Freie Universität Berlin, 14163 Berlin, Germany; Theresa.firsching@fu-berlin.de (T.C.F.); Achim.gruber@fu-berlin.de (A.D.G.),; 5 Public Health, Jockey Club College of Veterinary Medicine and Life Sciences, City University of Hong Kong, Kowloon, Hong Kong; Klaus.osterrieder@fu-berlin.de; 6 Department of Infectious Diseases and Respiratory Medicine, Charité Universitätsmedizin Berlin, 13353 Berlin, Germany; Leif-erik.sander@charite.de

**Keywords:** SARS-CoV-2, vaccine genes, dwarf hamster, animal model, adenoviral vectors

## Abstract

With the exception of inactivated vaccines, all SARS-CoV-2 vaccines currently used for clinical application focus on the spike envelope glycoprotein as a virus-specific antigen. Compared to other SARS-CoV-2 genes, mutations in the spike protein gene are more rapidly selected and spread within the population, which carries the risk of impairing the efficacy of spike-based vaccines. It is unclear to what extent the loss of neutralizing antibody epitopes can be compensated by cellular immune responses, and whether the use of other SARS-CoV-2 antigens might cause a more diverse immune response and better long-term protection, particularly in light of the continued evolution towards new SARS-CoV-2 variants. To address this question, we explored immunogenicity and protective effects of adenoviral vectors encoding either the full-length spike protein (S), the nucleocapsid protein (N), the receptor binding domain (RBD) or a hybrid construct of RBD and the membrane protein (M) in a highly susceptible COVID-19 hamster model. All adenoviral vaccines provided life-saving protection against SARS-CoV-2-infection. The most efficient protection was achieved after exposure to full-length spike. However, the nucleocapsid protein, which triggered a robust T-cell response but did not facilitate the formation of neutralizing antibodies, controlled early virus replication efficiently and prevented severe pneumonia. Although the full-length spike protein is an excellent target for vaccines, it does not appear to be the only option for future vaccine design.

## 1. Introduction

In December 2019, a novel and readily transmissible coronavirus was identified in Wuhan, China and was associated with the multi-faceted coronavirus disease 2019 (COVID-19), with symptoms ranging from asymptomatic to severe pneumonia with respiratory failure [1]. As soon as the high pandemic potential became evident, vaccine developers and manufacturers around the globe commenced fast-paced efforts to develop an efficient COVID-19 vaccine [2]. Based on existing knowledge on human coronaviruses, almost all vaccination concepts focused on the spike envelope glycoprotein as a SARS-CoV-2 specific vaccine antigen [3].

Recently, the occurrence and rapid spread of viral variants carrying mutations in the SARS-CoV-2 genome, particularly within the gene encoding the spike glycoprotein, have raised concern. Through systematic whole-genome sequencing in many countries and comparative analyses of several hundreds of thousands SARS-CoV-2 genomes, more than 40,000 sequence deviations (deletions, insertions, single nucleotide polymorphisms) compared to the reference genome of the virus originally isolated in China have been detected (https://bigd.big.ac.cn/ncov/variation/annotation, accessed on 15 November 2021). Interestingly, these mutations are not distributed evenly across the entire virus genome, but concentrate in hotspots [4]. Mutations in the gene encoding the spike glycoprotein and the nucleocapsid protein accumulate much more rapidly compared to other genes, with the former being selected more quickly in the population [4,5]. Point mutations affecting individual amino acids may be sufficient to enhance the binding properties of the spike protein and thus the infectivity of the virus, as evidenced by mutations that occurred in the B.1.1.7 lineage first detected in England [6]. The occurrence of mutations in the spike gene raises concerns regarding an ongoing virus evolution towards immune evasion, and particularly, reduced antibody-mediated defense. It should therefore be investigated whether other or additional SARS-CoV-2 antigens can be considered in future vaccines to alleviate the pressure on the spike protein as a single immunological target.

The SARS-CoV-2 genome encompasses approximately 30,000 nucleotides and contains twelve open reading frames (ORF). ORF1a and ORF1b cover 71% of the whole genome and code for polyproteins, which are post-translationally processed into 16 smaller proteins (nonstructural proteins NSP1–16), and mostly involved in virus replication [7]. The remaining 29% of the genome code for four structural proteins, the membrane protein (M), the nucleocapsid protein (N), the envelope protein (E) and the spike protein (S), which are interspersed with ORFs encoding accessory proteins [7].

The spike protein is 1273 amino acids in length, which corresponds to 12.7% of the SARS-CoV-2 proteome. It is an essential component in the infection process, as it protrudes from the viral surface and initiates infection of human cells by binding to the angiotensin converting enzyme receptor 2 (ACE2) [8]. Blocking the receptor binding domain (RBD) of the spike protein by neutralizing antibodies efficiently inhibits infection [9]. All FDA and EMA approved SARS-CoV-2 vaccines contain full-length S as the vaccine antigen, and elicit both high levels of neutralizing antibodies and robust T-cell responses [10]. The RBD is mainly targeted by antibodies, whereas T-cell epitopes are mainly contained in regions outside of the RBD [11,12,13]. The respective contributions of humoral and cellular immune responses to vaccine efficacy are currently largely unclear.

The nucleocapsid protein is packing the viral RNA and is not present on the virion’s surface. Thus it does not induce the formation of high levels of neutralizing antibodies. This suggests that any protective immune response against N would likely be mediated by the formation of specific cytotoxic T-cells.

In this study, we compared the immunogenicity and efficacy of different SARS-CoV-2 vaccine antigens, including RBD, full-length spike, a combination of RBD and M, as well as N, all encoded by a replication deficient human adenovirus serotype 2 (Ad2). Due to its high susceptibility to severe COVID-19-like disease, we used Roborovski dwarf hamsters in this study to mimic disease in highly susceptible patients.

## 2. Material and Methods

### 2.1. Cloning, Generation and Purification of Adenoviral Vaccines

The DNA synthesis of SARS-CoV-2 genes (nucleocapsid (N), membrane (M), full spike (S) and receptor binding domain (RBD, aa 277–588) was commissioned by gene art (ThermoFisher Scientific, Regensburg, Germany), according to the NCBI reference genome sequence (NC_045512.2). The codon was optimized and 3′ prime HA-tagged genes under the control of the CMV-promoter were cloned into an adenoviral backbone plasmid carrying the E1/E3 deleted genome of human adenovirus serotype 2 (pAd2-long) via homologous recombination. In the double gene vector, expression of the RBD was driven by the CMV-promoter and the membrane protein by the EMCV-IRES (CMV-RBD-IRES-membrane). The identity of cloned SARS-CoV-2 transgenes was confirmed by immunofluorescence staining and flow cytometric analysis (MACSQuant Analyser, Miltenyi Biotec Inc., Bergisch-Gladbach, Germany) and by Western blot analysis (Supplement) after transient transfections of expression plasmids and recombinant viruses on 293 and HeLa cells. Employed antibodies: FITC anti-SARS-CoV-2-spike glycoprotein (Biozol, Eching, Germany, ABA-AB02019–23-FITC), FITC anti-SARS-CoV-2 nucleocapsid protein (Biozol, Eching, Germany, ABA-AB01691–23-FITC), anti-SARS-CoV-2 membrane protein (Biozol, Eching, Germany, PRS-10–516-0.1) and anti-HA-HRP (Miltenyi, Bergisch-Gladbach, Germany, 130–091-972).

For generation of recombinant adenoviral vaccines, purified plasmid DNA was transfected on 293 cells and agarose overlay was subsequently performed. Seven days after transfection, the first virus plaques became visible. Single plaques were isolated and after second plaque purification, viruses were further propagated on 293 cells and harvested 3 days after infection. Cells were lysed by repeated freeze-thaw cycles and viral particles were purified by 2 rounds of cesium chloride density gradient centrifugation. Cesium chloride was removed by gel filtration on Sephadex G-25 (Pharmacia, Uppsala, Sweden). Equilibration was performed with injection buffer, containing 3 mM KCl, 1 mM MgCl2, phosphate buffered saline (PBS) and 10% glycerol. A 0.45 µm filter was used for sterile filtration (Schleicher und Schuell, FP 030/2, Dassel, Germany). The virus suspension was stored in aliquots at −78 °C. Titration was carried out by endpoint dilution assays on 293 cells in 96-well plates. In addition, particles were counted, using the spectrophotometry technique described previously [14]. Biologic titers were expressed as infectious units (IFU) per mL and physical titers as particles/mL. Particle concentrations of adenoviral stock solutions ranged from 2–5 × 10^11^ particles/mL.

### 2.2. SARS-CoV-2 Virus and Cells

The virus isolate BetaCoV/Germany/BavPat1/2020 [15] T-cell culture passage 3 was used for SARS-CoV-2 animal challenge. The virus was propagated and titrated on Vero E6 cells (ATCC CRL-1586) in minimal essential medium (MEM; PAN Biotech, Aidenbach, Germany) supplemented with 10% fetal bovine serum (PAN Biotech, Aidenbach, Germany), 100 IU/mL penicillin G and 100 µg/mL streptomycin (Carl Roth, Karlsruhe, Germany), and stored at −80 °C prior to experimental infections. Genome integrity, specifically the presence of the furin cleavage site, was confirmed by NGS sequencing of virus stocks used for animal experimentation.

### 2.3. Animals and Vaccination

Animal procedures were performed according to the European Guidelines for Animal Studies after having been approved by the Institutional Animal Care Committee and the relevant state authority (Landesamt für Gesundheit und Soziales, Berlin, Permit number 0086/20). In each group, nine male and female Roborovski dwarf hamsters (*Phodopus roborovskii*) obtained via the German pet trade were used. Animals were housed in groups of 3–6 hamsters in GR-900 IVC cages (Tecniplast, Buguggiate, Italy) and provided with bountiful enrichment and nesting materials (Carfil, Oud-Turnhout, Belgium). Hamsters were randomly distributed into experimental groups and individually marked with a subcutaneously implanted IPTT-300 transponder (BMDS, Seaford, DE, USA) that allows remote identification and measurement of body temperature. All hamsters were vaccinated by a single intraperitoneal application of 5 × 10^8^ infectious units (IFU) in 200 µL injection buffer of adenoviral SARS-CoV-2 vaccines or a control adenovirus vector (Ad-LacZ). Previous studies as well as own observations have shown that there is no significant difference regarding the induced immune response between the intraperitoneal and intramuscular rout of application [16,17]. However, intraperitoneal injection is less stressful for the animals and allows a more reliable and precise dosing.

C57BL/6 mice were purchased from Charles River (Sulzfeld, Germany). In total, 5 groups each of 5 animals were set up and immunization was performed by intraperitoneal application of 5 × 10^8^ i.p. Ad2-spike, Ad2-RBD, Ad2-RBD+membrane, Ad2-nucleocapsid and Ad-LacZ (Ad-mock). Three weeks after immunization animals were sacrificed, blood was taken and spleens were removed for performing virus neutralization tests and T-cell restimulation assays.

### 2.4. Infection Experiments

SARS-CoV-2 infection was performed as previously described [18]. Briefly, anesthetized hamsters received 1 × 10^4^ pfu SARS-CoV-2 in 20 µL MEM intranasally 21 days post vaccination with adenovirus constructs. Following infection, the clinical presentation of all animals was monitored twice daily, body weights and temperatures of all hamsters were recorded daily. On days 2, 5 and 7 post infection, three hamsters per group were euthanized, as either randomly assigned or based on defined score-sheet criteria for humane endpoints. Euthanasia was applied by exsanguination under general anesthesia as described [19]. Whole blood, serum, oropharyngeal swabs and lungs were collected for virus titrations, RT-qPCR and/or histopathological examinations. All organs were immediately frozen at −80 °C or preserved in 4% formalin for subsequent in-depth histopathological investigations.

### 2.5. Virus Titrations, RNA Extractions and RT-qPCR

To determine virus titers from 50 mg of lung tissue, tissue homogenates were prepared using a bead mill (Analytik Jena, Jena, Germany), and 10-fold serial dilutions were prepared in MEM, which were then added to Vero E6 cells in 12-well plates. The dilutions were removed after 2 h and cells were overlaid with 1.25% microcrystalline cellulose (Avicel, FMC BioPolymer, Hamburg, Germany) in MEM supplemented with 10% FBS and penicillin/streptomycin. Three days later, cells were formalin-fixed, stained with crystal violet and plaques were counted. RNA was extracted from 25 mg of lung homogenates and oropharyngeal swabs using the innuPREP Virus RNA kit (Analytik Jena, Jena, Germany). Viral RNA copies were quantified in 10% of the obtained eluate volume with a one-step RT-qPCR reaction using a standard curve and the NEB Luna Universal Probe One-Step RT-qPCR kit (New England Biolabs, Ipswich, MA, USA) and previously published TaqMan primers and probe (SARS-CoV-2 E_Sarbeco) [20] on a StepOnePlus RealTime PCR System (Thermo Fisher Scientific, Waltham, MA, USA).

### 2.6. Serum Neutralization Tests (SNT)

To determine the capacity of serum obtained from hamsters post and mice pre SARS-CoV-2 challenge to neutralize SARS-CoV-2 in vitro, we performed serum neutralization assays. Serum complement was inactivated for 30 min at 56 °C and prepared in duplicates as two-fold serial dilutions in MEM supplemented with 10% FBS and penicillin/streptomycin in 96-well cell culture plates (Sarstedt, Nümbrecht, Germany). To each serum dilution and the respective control wells, 40 pfu of SARS-CoV-2 was added and inactivation was allowed to proceed for 30 min at room temperature. Afterwards, approximately 1 × 10^4^ Vero E6 cells were added to each well. The plates were incubated at 37 °C under a 5% CO_2_ atmosphere for 3 days, fixed with 4% formaldehyde and stained with 0.75% crystal violet (aqueous solution) to determine cytopathic effects (cpe). Virus neutralization was considered successful in wells with no evidence of cpe, the last effective serum dilution was counted.

### 2.7. T-Cell Restimulation Assays and Flow Cytometry

Spleen were forced through 100 µm cell strainers (Falcon/Corning, Kaiserslautern, Germany) with 4 °C cold phosphate buffered saline (PBS, Gibco, Dreieich, Germany) and centrifuged at 4 °C and 300× *g*. Then, erythrocytes were lysed in hypotonic PBS diluted in a 1:3 ratio with sterile deionized H_2_O for 12 s. Lysis was stopped by addition of PBS supplemented with 2% (vol/vol) fetal calf serum (FCS, Biochrom AG, Berlin, Germany). Single cell suspensions were filtered through 40 µm cell strainers (Falcon/Corning, Kasierslautern, Germany) with 4 °C PBS containing 2% (vol/vol) FCS. Cells were re-stimulated with either a mixture of 11aa overlapping 15 mer PepMix^TM^ SARS-CoV-2 spike glycoprotein peptide pool 1 and a custom made peptide pool for CD8 T-cells (both from JPT Peptide Technologies GmbH, Berlin, Germany) or nucleocapsid peptide mix (peptides&elephants, Henningsdorf, Germany) at 1 μg/5 × 10^6^ cells in VLE RPMI 1640 with glutamine supplemented with 10% (vol/vol) FCS, 100 U/mL penicillin, 100 mg/mL streptomycin (all Biochrom AG, Berlin, Germany) and 50 µM β-mercaptoethanol (Sigma-Aldrich) for 16 h at 37 °C and 5% CO_2_. For cytokine detection, cells were treated prior to antibody staining with 2 µg/mL Brefeldin A (Sigma-Aldrich, Taufkirchen, Germany) for the last two hours.

Dead cells were stained using Zombie UV™ Fixable Viability Kit (Biolegend, San Diego, CA, USA, 1:100) according to manufacturer’s protocol and Fc receptors were blocked with purified rat anti-mouse CD16/CD32 Mouse BD Fc Block™ (2.4G2, BD Pharmingen, San Diego, CA, USA, 1:1000) in PBS. Cell surface antigens were stained in PBS containing 2% (vol/vol) FCS and 1 mg/mL sodium azide (Serva, Heidelberg, Germany) at 4 °C in the dark. Cell fixation and staining of intracellular antigens was completed with Foxp3 Staining Buffer Set (Miltenyi, Bergisch-Gladbach, Germany) for transcription factor staining or with Fixation/Permeabilization Solution Kit (BD, Heidelberg, Germany, Cytofix/Cytoperm) for cytokine staining according to manufacturer’s protocols. Monoclonal antibodies against the following antigens were used: CD4 (RM4–5, Biolegend, San Diego, CA, USA), CD8a (53-6.7, Biolegend), CD44 (IM7, Biolegend), CD62L (MEL-14, Biolegend), GZMB (QA16A02, Biolegend), IFN-γ (XMG1.2, Biolegend), Ki67 (SolA15, eBioscience, San Diego, USA), TCRβ (H57-597, Biolegend), KLRG1 (2F1/KLRG1, Biolegend), CD127 (A7R34, Biolegend). Data were acquired using an LSRFortessa with BD FACSDiva software v8.0.2 (BD Biosciences) and analyzed with FlowJo v10 software (FlowJo, LLC, Ashland, OR, USA).

### 2.8. Histopathology

Histopathology was performed as described in [21] on formalin-fixed, paraffin embedded entire left lungs at two separate, representative planes across the hilum using hematoxylin and eosin stained sections of 2 μm. Pulmonary lesions were scored according to standardized reporting criteria for SARS-CoV-2 infected hamsters [22], with score values ranging from 0 to 3 (0: no lesions; 1: mild; 2: moderate; 3: severe).

### 2.9. Statistics

Flow cytometry data were displayed as boxplots with 5–95 percentile whiskers. Statistical significance was determined as indicated in the figure legend. Datasets were analyzed by Kruskal–Wallis test followed by post hoc Dunn’s multiple comparison test. P-values below 0.05 were considered as statistically significant. Statistical analysis was conducted using GraphPad Prism v9 software (GraphPad Software).

## 3. Results

### 3.1. Course of Body Weight and Temperature after SARS-CoV-2 Exposure

To explore the immune protective potency of different SARS-CoV-2-specific vaccine antigens, we generated recombinant replication deficient adenoviral vectors (del. E1/E3 Ad2) encoding full-length spike (S), receptor binding domain (RBD), nucleocapsid protein (N) and dual antigen expressing the receptor binding domain (RBD) and the membrane protein (Roborovski dwarf hamsters were vaccinated three weeks prior to challenge with wild type SARS-CoV-2). Temperature and body weight were recoded throughout the challenge experiment. SARS-CoV-2 virus load was assessed both as replication competent virus in the lungs of infected animals and genomic RNA copies in the throat and lungs. Additionally, neutralizing antibody titers were determined, and histological evaluation of lung tissue was performed on day 2, 5 and 7 days after viral challenge. Roborovski dwarf hamsters are very sensitive to SARS-CoV-2 infection and develop severe COVID-19-like disease within the first days following infection [18]. After intranasal inoculation with 1 × 10^4^ plaque-forming units (PFU) of wild type SARS-CoV-2, virus loads quickly increased in the throat and lungs of infected animals. Forty-eight hours after infection, virus loads in the lungs of unvaccinated animals increased to on average approximately 10^7^ SARS-CoV-2 genome copies.

**Figure 1:**
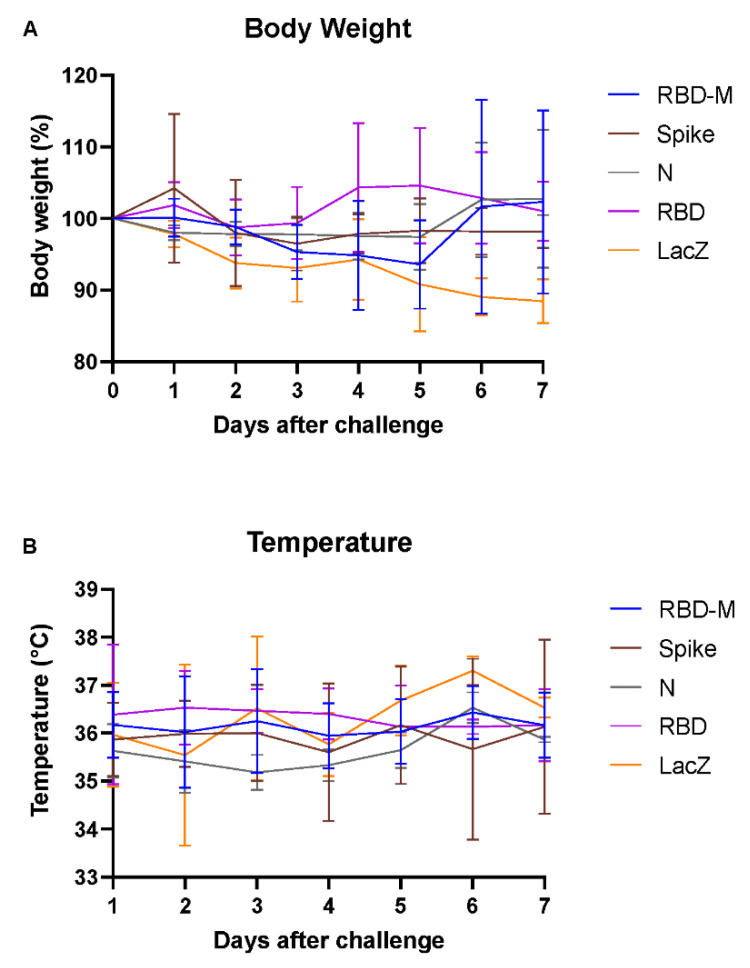
Changes in body weight and temperature in Roborovski dwarf hamsters vaccinated with different adenovirus vector vaccines (RBD-M, Spike, N, RBD, Lac Z) following SARS-CoV-2 challenge. (**A**) Body weight changes for hamsters vaccinated with adenovirus vector vaccines containing different SARS-CoV-2 transgenes, shown as group mean with SD over the course of 7 days after infection (per group, *n* = 9 until day 2, *n* = 6 after 2 dpi and *n* = 3 after 5 dpi). The color-coding represents different transgenes: RBD-M (blue), Spike (red), N (green), RBD (purple) and LacZ (orange). (**B**) Temperature changes shown as group mean with SD over the course of 7 days after infection (per group, *n* = 9 until day 2, *n* = 6 after 2 dpi and *n* = 3 after 5 dpi). The color-coding represents different transgenes: RBD-M (blue), Spike (red), N (green), RBD (purple) and LacZ (orange).

Application of Ad2-based SARS-CoV-2 vaccines was well tolerated in all Roborovski dwarf hamsters. No adverse reactions were observed in any vaccinated animal throughout this study. Upon challenge with SARS-CoV-2, none of the animals reached a defined humane endpoint by day 7 after challenge. However, body weights in the Ad-lacZ control group dropped by 10% at day 7 compared to the pre-infection weight, while no clinical differences were observed between groups that had received vaccines containing SARS-CoV-2 antigens (Figure 1A). Body temperatures of all animals remained stable at levels typical of this hamster species. A drop in temperature indicative of a severe course of infection [18] was not observed (Figure 1B).

### 3.2. Infectious SARS-CoV-2 Viruses and RNA Copies Isolated from Lung and Throat Swabs

**Figure 2:**
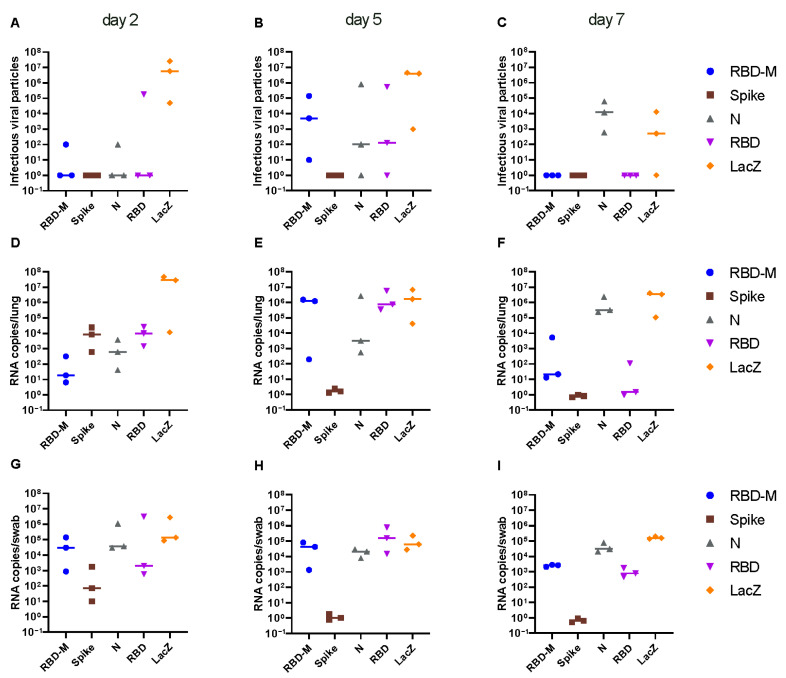
Infectious SARS-CoV-2 particles in lungs (**A**–**C**) and virus RNA copy numbers isolated from lungs (**D**–**F**) and from throat swabs (**G**–**I**) of Roborovski dwarf hamsters 2, 5 and 7 days after challenge with SARS-CoV-2 wild type virus. Three weeks prior to challenge, hamsters had received a single application of adenoviral vaccines encoding the full-length spike protein (S, spike), the receptor binding domain (RBD), the nucleocapsid protein (N), a double gene vector encoding the receptor binding domain (RBD) and the membrane protein (M) and a control vector encoding bacterial ß-galactosidase (LacZ). Virus titers in 50 mg lung tissue were determined by plaque assay on Vero E6 cells. Virus genome copy numbers per 2.5 mg lung tissue and in oropharyngeal swabs as determined by RT-qPCR.

Replication-competent viral particles were quantified in lung homogenates. Vaccination with full-length spike encoding vaccine provided complete protection from viral replication in the lung of all animals at all time points post challenge, indicating robust sterilizing immunity elicited by this vaccine (Figure 2A–C). A drastic reduction in virus titers in the lung was achieved with all SARS-CoV-2 specific vaccines, regardless of the encoded antigen at the early time point (day 2) post challenge (Figure 2A). At later time points (day 5 + 7), only full-length spike provided complete protection, whereas all other vectors achieved partial protection from lung infection (Figure 2B,C). Animals vaccinated with Ad2-RBD or Ad2-RBD-M cleared the lung infection by day 7 post challenge, while Ad2-N vaccinated animals failed to control the infection at day 7, despite early reduction of viral loads (Figure 2B,C). Similar results were obtained when quantifying genomic RNA (gRNA) in the lungs. In contrast to lung infection, only Ad2-S elicited sterilizing immunity in the throat, whereas all other vaccines failed to control viral loads in the upper respiratory tract (Figure 2A–C).

### 3.3. Histopathology of Lung Tissue after Infection with SARS-CoV-2

**Figure 3:**
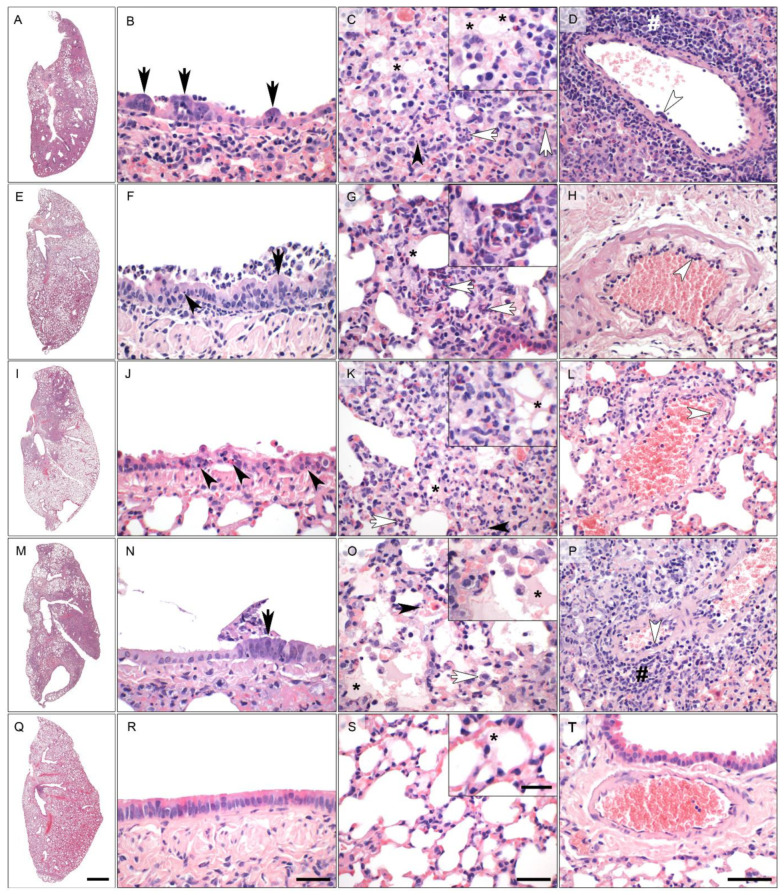
Representative histopathology (hematoxylin and eosin stain) of the left lung of vaccinated Roborovski dwarf hamsters 5 days after challenge with wild type SARS-CoV-2 viruses. (**A**–**D**) **(RBD + membrane protein group):** the overview of the left lung revealed extensively affected areas (**A**). The bronchiolar epithelium (**B**,**E**) had markedly hyperplastic bronchiolar epithelial cells (**F**; black arrows) and cellular debris inside the bronchiolar lumen (**B**). The alveoli were characterized by marked diffuse alveolar damage with neutrophils, macrophages (white arrows) and necrosis of alveolar epithelial cells (black arrowhead). Alveolar edema was mild to moderate (asterisk) (**C**). Blood vessels of hamsters treated with Adeno A occasionally presented with endothelialitis (white arrowhead) and perivascular lymphocytic cuffing (hash) (**D**). (**E**–**H**) **(Nucleocapsid group):** similar to the hamsters of the full spike protein group, hamsters treated with the nucleocapsid protein had only small lung areas affected by inflammatory changes (**E**). However, bronchioles were multifocally characterized by mild to moderate necrosuppurative bronchiolitis and signs of regeneration (black arrow); necrosis of BEC (black arrowhead) (**F**). The few areas with interstitial pneumonia in the lungs of nucleocapsid group of hamsters presented with influx of neutrophils and macrophages (white arrows, inset) and mild alveolar edema (asterisk) (**G**). Blood vessels showed only very mild evidence of endothelialitis (white arrowhead) (**H**). (**I**–**L**) **(Receptor binding domain group):** approximately half of the left lungs was affected in hamsters vaccinated with the RBD vaccine (**I**). (**B**,**E**) was characterized by moderate to severe bronchiolitis with marked necroses of BEC (black arrowheads) and regeneration (not depicted) (**J**). Moderate to severe interstitial pneumonia with neutrophils and macrophages (white arrow) but also necroses (black arrowhead) and alveolar edema (asterisk, inset) was observed (**K**). Mild to moderate vascular lesions with endothelialitis (white arrowhead) were also present (**L**). (**M**–**P**) **(Bacterial ß-galactosidase):** more than half of the left lung was affected in the majority of hamsters treated with the control vaccine (LacZ) (**M**). Bronchioles were characterized by moderate necrosuppurative bronchiolitis with hyperplasia of BE (black arrow) (**N**). Moderate but diffuse (see **Q**) interstitial pneumonia with neutrophils, macrophages (white arrow) and necrosis of alveolar epithelial cells (black arrowhead) was observed. Alveolar edema was mild to moderate (inset, asterisk) (**O**). Blood vessels had moderate endothelialitis (white arrowhead) and mild perivascular lymphocytic cuffing (hash) (**P**). (**Q**–**T**) **(Full spike protein group):** hamsters pre-treated with an adenovirus encoding the full spike protein presented with a small affected lung area (**Q**). The BE showed no lesions after infection with SARS-CoV-2 (**R**). In the alveoli, only mild interstitial pneumonia with influx of few neutrophils and macrophages as well as minimal alveolar edema (inset, asterisk) were observed. Necrosis was not a histological feature in this group. (**S**). Vascular injury was absent from the majority of blood vessels of hamsters pre-treated with full length spike-encoding vector vaccine (**T**). Scale bars: overview (**A**,**E**,**I**,**M**), 1 mm; central columns (**B**,**C**,**F**,**G**,**J**,**K**,**N**,**O**,**R**,**S**), 50 μm; insets, 20 μm; right column (**D**,**H**,**L**,**P**,**T**), 100 μm.

**Figure 4:**
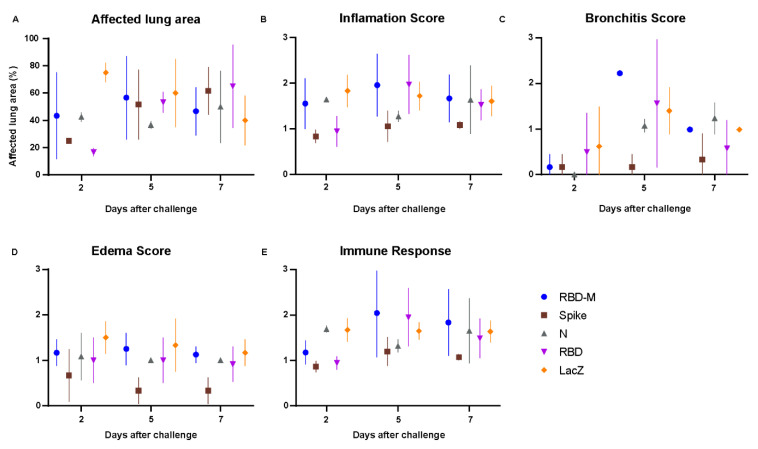
Inflammation score of lung histopathology in hamsters after SARS-CoV-2 infection. (**A**–**E**) Histopathological examination and pathological scoring of the lungs. (**A**) Estimate of the affected lung area in percent, (**B**) inflammation score, (**C**) bronchitis score, (**D**) edema score and (**E**) immune response score. Scores and parameters in graphs (**B**–**E**) were classified as absent (0), minimal (1), moderate (3) or severe (4).

In order to further assess protection, we analyzed infection-induced tissue damage by histopathology. We observed histological lesions consistent with previously described pathologies of SARS-CoV-2 infected Roborovski dwarf hamsters [22], however, with varying degrees between the experimental groups. Lungs from animals vaccinated with RBD-M, RBD and LacZ displayed overall strong inflammatory tissue damage, peaking at 5 and 7 dpi (Figure 3 and Figure 4). In comparison, the nucleocapsid (N) group at day 2 and 5 post infection was only mildly to moderately affected by inflammatory lesions of the same character. However, the lungs of animals at day 7 did not differ in terms of severity from the other three groups mentioned. Of note, few lungs of the RBD-M (1/3), N (2/3) and RBD (1/3) groups showed prominent infiltration by plasma cells at day 7. Inflammatory patterns of these four groups were dominated by bronchitis, pneumonia, necrosis of bronchiolar epithelial cells (BEC) and alveolar epithelial cells (AEC), as well as an influx of macrophages, neutrophils and fewer lymphocytes, accompanied by hyperplasia of type II AEC (Figure 3A–P). Perivascular and alveolar edema were also prominent. In sharp contrast, lungs of hamsters vaccinated with Ad2-S showed only very mild bronchointerstitial pneumonia, with little influx of macrophages and few neutrophils and lymphocytes at all time points observed (Figure 3Q–T).

### 3.4. Inflammation Score of Lung Histopathology

The majority of lungs in this group had virtually no bronchiolar (6/9), alveolar (5/9) or vascular (8/9) lesions. These results demonstrate that only Ad2-S provided full protection, while AdV-N provided partial protection from SARS-CoV-2 induced pneumonia (Figure 4).

### 3.5. Course of Serum Neutralizing Antibody Titers after SARS CoV-2 Challenge

To better understand the differential levels of protection afforded by the different vaccines, we compared immunogenicity and measured neutralizing antibody responses. Early after SARS-CoV-2 challenge, we performed serum neutralization tests (Table 1). We observed clear differences in neutralizing antibody levels on day 2 post-infection. While the RBD-M construct induced neutralizing antibody titers similar to the full-spike construct, RBD alone induced a lower neutralization capacity. As expected, no major neutralizing antibody response was observed in animals that had received Ad2-N or the Ad2-LacZ construct. By day 5 post-infection, differences between animals immunized with Ad2-S, Ad2-RBD-M and Ad2-RBD were not obvious, but those that had received the Ad2-N vaccine on average mounted a lower neutralizing response than animals of the Ad2-LacZ control group. On day 7 post-infection, all animals presented high neutralizing antibody titers indicative of protective immunity elicited by virus challenge.

In order to investigate the speed of antibody formation after exposure to SARS-CoV-2 titer determinations were carried out 2, 5 and 7 days post infection. It turns out that even the two groups (Ad-lacZ, Ad-N) that had no pre-existing antibodies at the time of first contact with SARS-CoV2 rapidly mounted an strong antibody response and showed high titers on day 7 already. Comparing the other three groups (Ad-RBD, Ad-S, Ad-RBD-IRES-M) demonstrates that the sole expression of the RBD does not stimulate the formation of NSTs as strong as the expression of full length spike. However, this deficit could be partially compensated by co-expression of the RBD with the membrane protein, which suggest a role for the membrane protein in antibody formation a well. 

### 3.7. Comparison of T-Cell Reactivity in Mice after Vaccination with Ad-S, Ad-N and Ad-RBD

**Figure 5:**
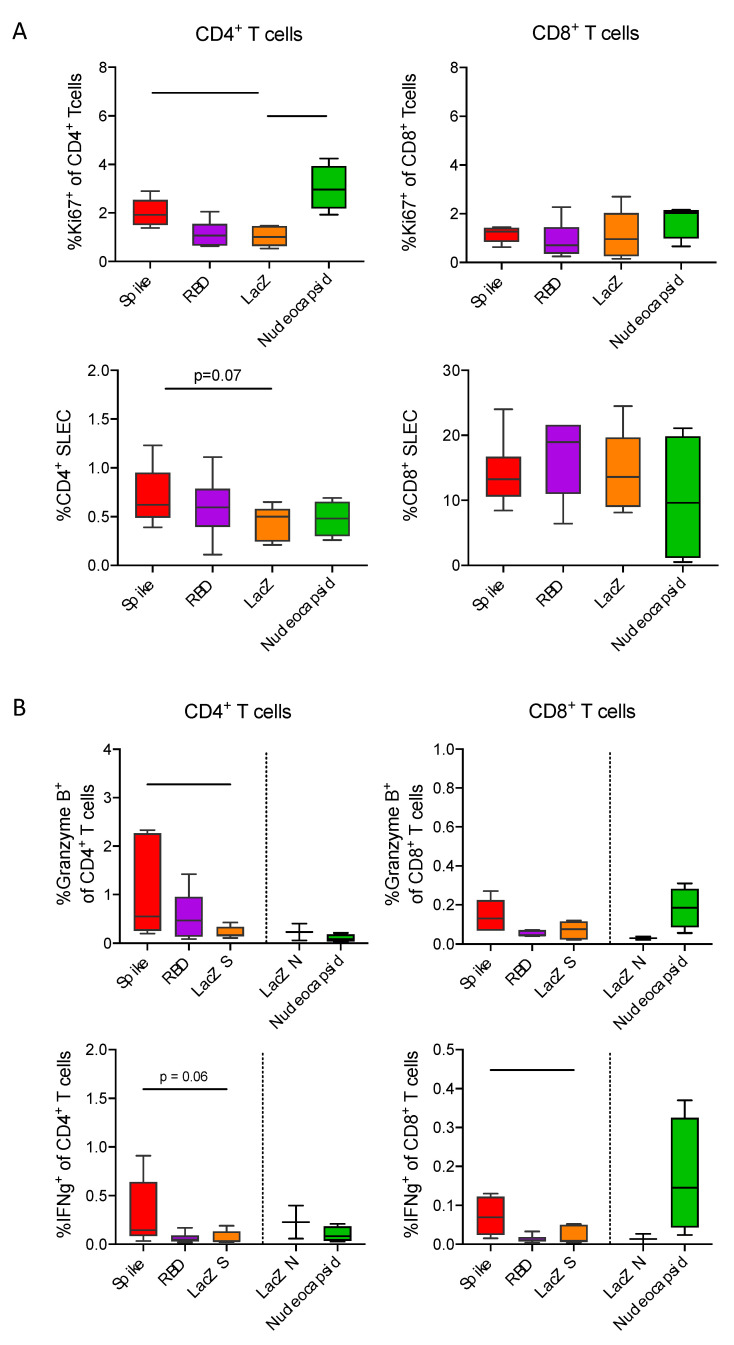
Full-length spike and nucleocapsid vaccines induce IFNg producing CD4^+^ respective CD8^+^ T-cell responses. (**A**) Boxplots with 5–95 percentile whiskers showing proportions of Ki67^+^ as well as CD44^+^KLRG1^−^CD127^−^ short-lived effector CD4^+^ and CD8^+^ T-cells in spleens collected three weeks after vaccination with adenovirus encoding full-spike (*n* = 6), RBD (*n* = 6), LacZ (*n* = 6) or nucleocapsid (*n* = 3).(**B**) Boxplots with 5-95 percentile whiskers showing proportions of CD4^+^ and CD8^+^ T cells expressing granzyme b or IFNg upon 16-hour restimulation with spike 1 peptide mixes (full spike *n* = 6, RBD *n* = 6, LacZ *n* = 5) or nucleocapsid peptide mix (nucleocapsid *n* = 3, LacZ *n* = 2). Data were analyzed by Kruskal-Wallis test followed by posthoc Dunn’s multiple comparison test. * *p* < 0.05, ** *p* < 0.01.

To better define immune responses to the different vaccines, we investigated antigen-specific CD4^+^ helper and CD8^+^ cytotoxic T-cell responses. Given the limited availability of tools and reagents to study T-cell responses in Roborovski dwarf hamsters, we performed immunizations in C57BL/6 mice. Three weeks after intra-peritoneal immunization with Ad2-S, Ad2-RBD or Ad2-N, we assessed proportions of proliferating and short-lived effector CD4^+^ and CD8^+^ T-cells in spleens (Figure 5A). Immunization with Ad2-S or Ad2-N induced increased levels of Ki67^+^ proliferating CD4^+^ T-cells as compared to the LacZ control. No such increase was detectable for CD8^+^ T-cells. In addition, vaccination with Ad2-S vaccine resulted in a trend towards higher proportions of CD44^+^KLRG1^+^CD127^−^ short-lived effector CD4^+^ T-cells. The higher proportion of SLEC CD4+ T-cells most likely reflected induction of SARS-CoV-2-specific immunity, as only upon vaccination with Ad2-S was there a significant increase detectable in Granzyme B^+^ and IFNg^+^ CD4^+^ T-cells upon peptide stimulation (Figure 5B). Consistent with our expectations, we observed an induction of SARS-CoV-2-specific Granzyme B^+^ and IFNg^+^ CD8^+^ T-cells upon vaccination with AdV-S or AdV-N.

## 4. Discussion

COVID-19 vaccines in clinical use activate both the humoral and the cellular arm of the immune system, thus leading to parallel formation of neutralizing antibodies and T-cell responses against the spike protein of SARS-CoV-2 [23]. Although clinical data confirm high efficacy for most vaccines, the contribution of cellular and humoral responses to the levels of protective immunity remains unclear. In this study, we compared the strength of vaccine-induced protection of four different adenovirus-based SARS-CoV-2-specific vaccines in hamsters, and explored the level of humoral response to challenge as well as the kinetic of virus clearance from the throat and lungs. By histopathology, signs of inflammation 2, 5 and 7 days after exposure to SARS-CoV-2 wild type virus were assessed. To better understand differences in immune responses, we additionally determined vaccine-induced T-cell responses in mice.

By far the strongest immune protection was achieved using a vector expressing the full-length spike protein, which blocked SARS-CoV-2 virus replication in the lungs by nearly 100% at all three time points explored in this study. The level of protection mediated by the other three constructs (RBD, RBD-IRES-membrane (M), nucleocapsid (N)) were mostly similar on day 2 and 5, while on day 7 the nucleocapsid-based vaccine showed a reduced ability to clear the virus from lung tissue. Through formation of neutralizing antibodies, it was expected that the RBD- and the RBD-IRES-membrane vaccine would be able to control early SARS-CoV-2 virus replication. The finding that the nucleocapsid protein-based vaccine, which evidently did not induce the formation of neutralizing antibodies, also blocked early viral replication but showed weak protection on day 7, and delayed antibody response at day 5 post infection was unexpected. We attribute this phenomenon to a partial control of SARS-CoV-2 replication by cellular immunity against the N protein, which was however not sufficient to eradicate the virus and thus allowed persistence of virus replication at later time points.

Two days after intranasal SARS-CoV-2 infection, an average of 5 million infectious particles were isolated from lung tissues of control animals (Ad-lacZ vaccinated), while in all other groups including the Ad-N group, the viral load was below 100. This finding suggests that in the absence of neutralizing antibodies, the cellular immune system alone is able to inhibit early virus replication very efficiently. This is further supported by our T-cell studies in mice, which revealed a particularly strong activation of cytotoxic T-cells after immunization with Ad-N (Figure 5).

In the Ad-N group, an efficient control of early viral replication is also indicated indirectly by the slower increase in neutralizing antibodies observed in this group (Table 1). In the control group (Ad-lacZ), 5 days after SARS-CoV-2 challenge, the physiological B-cell response had already yielded neutralizing antibody titers against SARS-CoV-2 of 1/65–1/256. At the same time, neutralizing antibodies in the Ad-N group were four steps lower and ranged between 1/8–1/16. This difference is possibly related to an efficient suppression of early viral replication, which leads to a slower accumulation of viral antigens and therefore to reduced immune response.

However, all hamsters with neutralizing antibodies at the time of challenge had cleared the virus from their lungs on day 7, while in hamsters immunized with Ad-N and with Ad-lacZ, an average of around 10^4^ infectious particles could still be isolated from lung tissue.

Coronaviruses replicate quickly and virus progeny are released from infected cells, starting 8 h after infection [18]. The outcome of the animal experiment suggests that at an early stage of the infection, as long as the viral load in the body is still low, the cellular immune system seems to be able to control the infection also in absence of neutralizing antibodies. However, as virus replication progresses, humoral immunity may become more important. Clearance of the infection by day 7 was only achieved by animals that had received a vaccine that did induce formation of neutralizing antibodies.

The relevance of high titers of neutralizing antibodies at the time of first contact with a coronavirus is also suggested from clinical observations, which show that vaccinated individuals who get infected with new SARS-CoV-2 variants which are less sensitive to neutralizing antibodies are at a higher risk of developing disease symptoms and shedding infectious virus, but only rarely develop a severe course of infection [24].

The ability of a COVID-19 vaccine to induce the formation of neutralizing antibodies is important. Our adenoviral constructs differ in terms of their ability to evoke neutralizing antibody response. The sole expression of the receptor binding domain (RBD, amino-acids 277–588 of spike) in hamsters only causes about 10% of the neutralizing antibody titers that are achieved after using the complete spike protein (Table 1). Likewise, the vaccine-induced T-cell response is significantly lower (Figure 5), indicating that epitopes for neutralizing antibodies and epitopes for CD8-T-cell activation are located outside the receptor binding region. Interestingly, the double gene vector (Ad-RBD-IRES membrane) encoding the RBD and the membrane protein induced more than a 10-fold higher neutralizing antibody titers compared to the sole expression of the RBD (Table 1), which suggests the need to further explore the role of the membrane protein for humoral immunity.

In summary, our study confirms the excellent immune protective properties of the full-length spike glycoprotein of SARS-CoV-2. Compared to this, the sole use of the RBD seems to provide a lower B- and T-cell antigenicity and less effective immune protection. The membrane protein might contribute to the formation of neutralizing antibodies as well, which should be explored further and might become useful for future vaccine design. In addition, our data suggest a role of the cellular immune system in preventing disease and controlling virus replication at an early phase of infection. In this resgard, the nucleocapsid protein might become an interesting candidate for future vaccines as well. Despite the small size (419 aa), it mediates a particularly strong SARS-CoV-2-specific cellular immune response, which might help to expand the antigenic basis of vaccine-induced immunity. Novel SARS-CoV-2 variants remain a constant threat to the protectivity of currently used vaccines. A more diverse selection of SARS-CoV-2 antigens used in vaccines could strengthen the vaccination campaign by making it more difficult for novel virus variants to undermine vaccine-induced immune protection. This could also alleviate the need for adjusting purely spike-based vaccines to the great diversity of circulating spike variants.

## Figures and Tables

**Table 1:** Serum neutralization titers for SARS-CoV-2 in Roborovski dwarf hamsters vaccinated with different adenovirus vector vaccines (RBD-M, Spike, N, RBD, Lac Z) on day 2, 5 and 7 after coronavirus challenge. Shown is the maximal serum dilution that still caused complete neutralization of authentic SARS-CoV-2 in a cell culture assay.

Vaccine Gene	day 2	day 5	day 7
N	0 / 0 / 8	8 / 8 / 16	256 / 512 / 512
RBD	16 / 32 / 32	256 / 512 / 512	512 / 512 / 512
S	256 / 512 / 512	512 / 512 / 512	512 / 512 / 512
RBD-IRES-M	256 / 256 / 512	64 / 512 / 512	512 / 512 / 512
mock-lacZ	0 / 0 / 4	64 / 64 / 256	512 / 512 / 512

## Data Availability

Data are contained within the article or Supplementary Material.

## Supplementary Materials

The following are available online at https://www.mdpi.com/article/10.3390/v13112290/s1, Figure S1: Protein expression patterns (western blotting, reducing conditions) 48 hours after after transient transfection of adenoviral vectors (Ad2-lacZ, Ad2-RBD-IRES-M, Ad2-S, Ad2-N, Ad2-RBD) on 1 × 10^6^ hela cells (MOI 50). Detection of HA-tagged antigens were performed by anti-HA-HRP (Miltenyi, Bergisch-Gladbach, Germany, 130-091-972) and visualised by ECL-chemiluminescence. Negative control: Ad-lacZ. The Membrane protein (M) has a calculated molecular weight of 25.2 kd and shows two bands at 27 and 20 kDa (10% gel). Full length spike (S) has a calculated molecular weight of 141.2 kd and shows two bands at 180 and 90 kDa. The Nucleocapsidprotein (N) has a calculated weight of 45.6 kd and shows three bands at 53, 47 and 45 kDa. The RBD has a calculated weight of 34.9 kd and shows two major bands at 39 and 35 kDa. The levels of antigen expression between S, N and the RBD are comparable, while the expression level of the membrane protein reaches only 20–30% compared to the other three antigens. M, S and the RBD are expressed under control of a CMV-promoter and the membrane protein via an ECMV-IRES.
