# Supplementary material for: Deciphering the Role of Humoral and Cellular Immune Responses in Different COVID-19 Vaccines—A Comparison of Vaccine Candidate Genes in Roborovski Dwarf Hamsters
Source: Viruses. 2021 Nov 16;13(11):2290. doi: 10.3390/v13112290 (PMC8625836; doi:10.3390/v13112290)
Supplement: Supplementary file 1 [file viruses-13-02290-s001.zip › viruses-1414328-supplementary.pptx]

## Slide 1
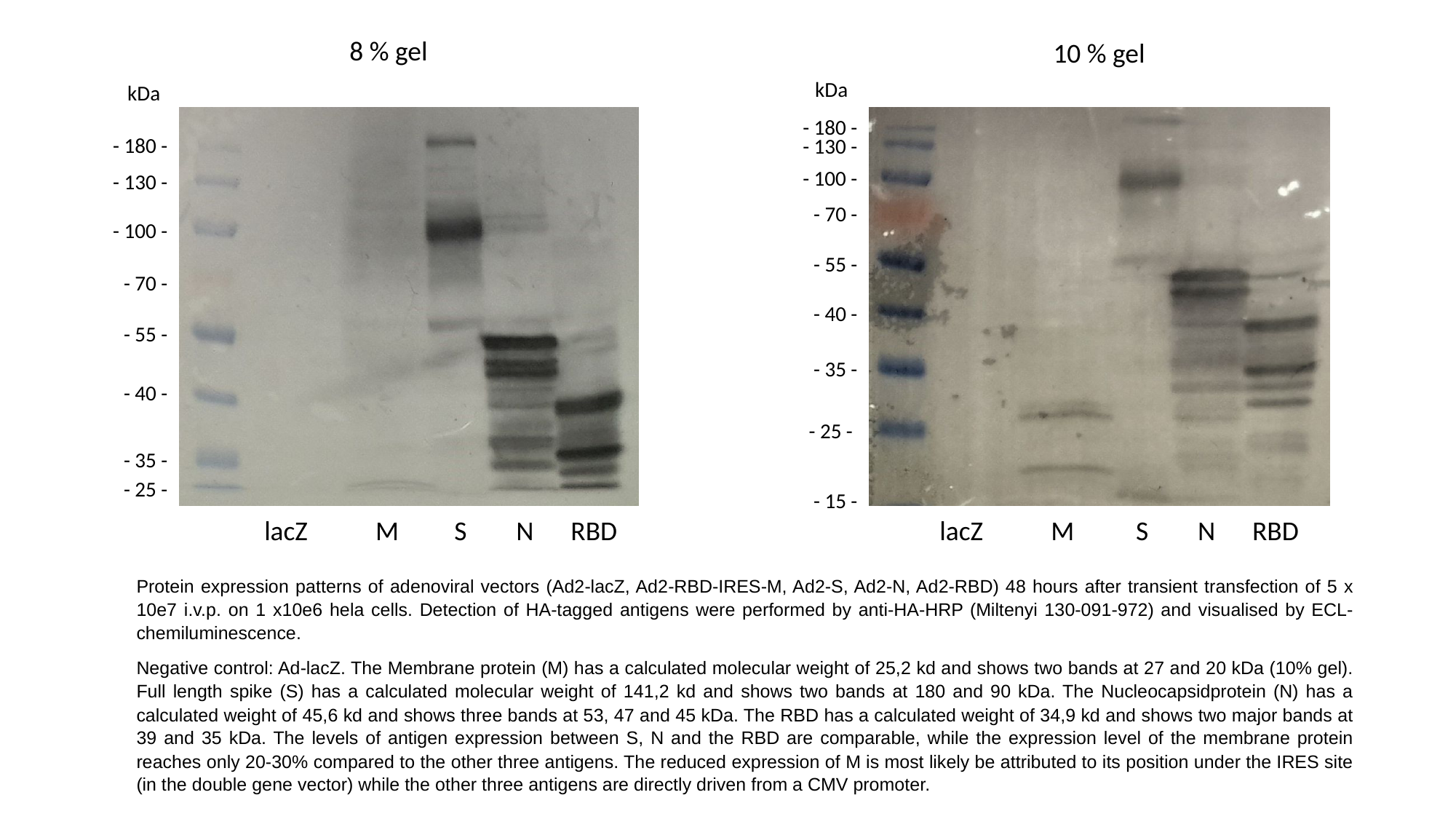

8 % gel
10 % gel
kDa
kDa
- 180 -
- 130 -
- 100 -
- 70 -
- 55 -
- 40 -
- 35 -
- 25 -
- 180 -
- 130 -
- 100 -
- 70 -
- 55 -
- 40 -
- 35 -
- 25 -
- 15 -
lacZ M S N RBD
lacZ M S N RBD
Protein expression patterns of adenoviral vectors (Ad2-lacZ, Ad2-RBD-IRES-M, Ad2-S, Ad2-N, Ad2-RBD) 48 hours after transient transfection of 5 x 10e7 i.v.p. on 1 x10e6 hela cells. Detection of HA-tagged antigens were performed by anti-HA-HRP (Miltenyi 130-091-972) and visualised by ECL-chemiluminescence.
Negative control: Ad-lacZ. The Membrane protein (M) has a calculated molecular weight of 25,2 kd and shows two bands at 27 and 20 kDa (10% gel). Full length spike (S) has a calculated molecular weight of 141,2 kd and shows two bands at 180 and 90 kDa. The Nucleocapsidprotein (N) has a calculated weight of 45,6 kd and shows three bands at 53, 47 and 45 kDa. The RBD has a calculated weight of 34,9 kd and shows two major bands at 39 and 35 kDa. The levels of antigen expression between S, N and the RBD are comparable, while the expression level of the membrane protein reaches only 20-30% compared to the other three antigens. The reduced expression of M is most likely be attributed to its position under the IRES site (in the double gene vector) while the other three antigens are directly driven from a CMV promoter.
